# How to Enhance the Power to Detect Brain–Behavior Correlations With Limited Resources

**DOI:** 10.3389/fnhum.2018.00421

**Published:** 2018-10-16

**Authors:** Benjamin de Haas

**Affiliations:** Experimental Psychology, Justus Liebig University Giessen, Giessen, Germany

**Keywords:** power, replication, individual differences, fMRI, MEG

## Abstract

Neuroscience has been diagnosed with a pervasive lack of statistical power and, in turn, reliability. One remedy proposed is a massive increase of typical sample sizes. Parts of the neuroimaging community have embraced this recommendation and actively push for a reallocation of resources toward fewer but larger studies. This is especially true for neuroimaging studies focusing on individual differences to test brain–behavior correlations. Here, I argue for a more efficient solution. *Ad hoc* simulations show that statistical power crucially depends on the choice of behavioral and neural measures, as well as on sampling strategy. Specifically, behavioral prescreening and the selection of extreme groups can ascertain a high degree of robust in-sample variance. Due to the low cost of behavioral testing compared to neuroimaging, this is a more efficient way of increasing power. For example, prescreening can achieve the power boost afforded by an increase of sample sizes from *n* = 30 to *n* = 100 at ∼5% of the cost. This perspective article briefly presents simulations yielding these results, discusses the strengths and limitations of prescreening and addresses some potential counter-arguments. Researchers can use the accompanying online code to simulate the expected power boost of prescreening for their own studies.

## Introduction

Recent estimates show that the statistical power of typical studies in neuroscience is inadequately low (Button et al., 2013). Low power and a publication bias for significant results lead to low replicability of published findings. Many researchers, journals and funding agencies are acutely aware of the problem and discuss a range of potential remedies, including the publication of data and analysis code (Pernet and Poline, 2015), preregistration (Chambers, 2013) and an increase of typical sample sizes (Dubois and Adolphs, 2016).

In parallel to this, cognitive neuroscience is gaining interest in individual differences revealing brain–behavior correlations (Kanai and Rees, 2011; Dubois and Adolphs, 2016). This trend includes subfields like visual neuroscience (Charest and Kriegeskorte, 2015; Genç et al., 2015; Moutsiana et al., 2016), which traditionally have treated such differences as ‘noise’ (Wilmer, 2008; Yovel et al., 2014; Peterzell and Kennedy, 2016). Studies investigating individual differences typically require larger sample sizes, and in light of the replicability debate, Dubois and Adolphs (2016) recently proposed a new standard of ‘*n* > 100.’

Here, I argue for a more efficient way of increasing the power to detect brain–behavior correlations. Simulations show that adequate power can be achieved with a prescreening approach, in which researchers test a larger sample behaviorally and selectively sample extreme-groups for brain scanning. Compared to the proposed increase in sample size, this approach typically enhances power at a fraction of the cost. Additionally, prescreening can ensure the reliability of measures, which is limiting observable effect sizes (see below).

Many others have discussed the importance of reliable measures (see below), as well as the advantages and limitations of prescreening (Alf and Abrahams, 1975; Abrahams and Alf, 1978; Preacher et al., 2005; Preacher, 2015). This perspective does not aim to make original points about either issue *as such*. Instead, it aims to highlight their special importance in studies using expensive techniques like MEG or MRI to study brain–behavior correlations. This boundary condition renders prescreening an efficient way to well-powered studies.

## Ensuring Reliability

The reliability of inter-individual differences in a given behavioral or neural measure depends on two sources. It increases with true between-subject variance of the measured trait and decreases with measurement error. Reliability can be estimated as the consistency between trials, items or parallel forms, or as test–retest reliability. As has been pointed out by others (Hedge et al., 2018), a given measure can robustly detect an effect at the group level, without reliably capturing individual differences. Personality and intelligence research have a history of developing reliable measures of behavioral variance (with the search for robust neural correlates of these measures proving more difficult; Yarkoni, 2015; Dubois et al., 2018). But as the investigation of brain–behavior correlations is adopted in other fields, the reliability of behavioral measures is often unknown *a priori*. Prescreening can be used to ensure and quantify this quality.

**FIGURE 1:**
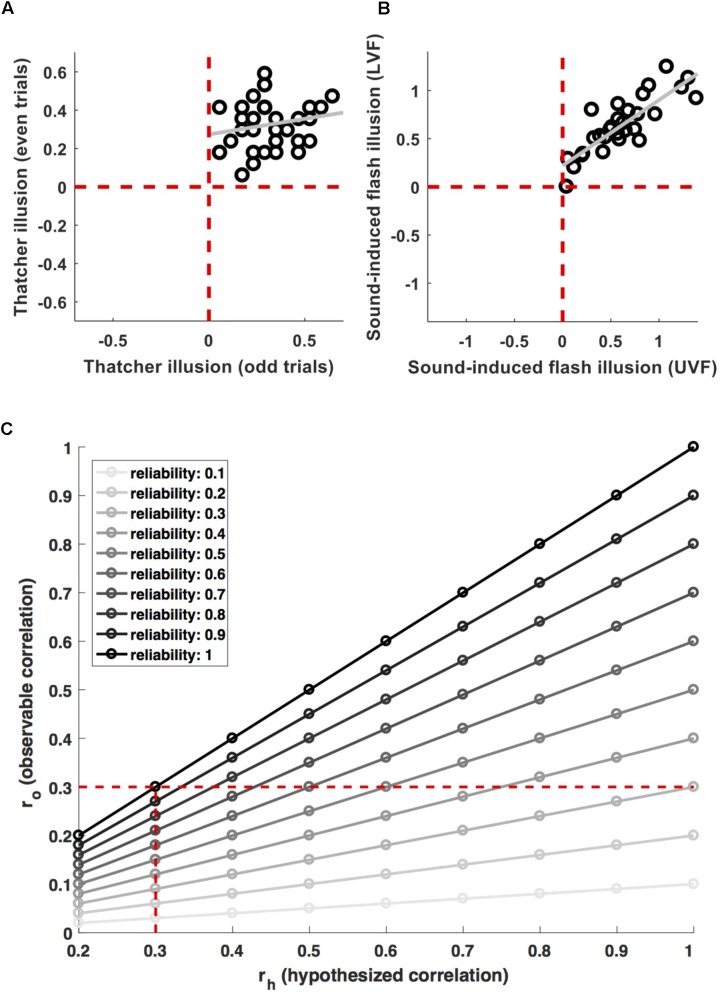
Measurement relibaility. **(A)** Shows individual data for the magnitude of the Thatcher illusion in odd and even trials from (de Haas and Schwarzkopf, 2018a). The effect is highly robust on the group level (all data are in the top right quadrant, i.e., every participant shows the effect in both, odd and even trials). But the inter-individual variance is inconsistent (*r* = 0.2). **(B)** Shows individual data for the magnitude of the sound-induced flash illusion in the upper (UVF) and lower (LVF) visual field from (de Haas et al., 2012). Here, the group-level effect as well as the inter-individual variance proved robust (*r* = 0.84). **(C)** Shows the attenuation of observable correlations (*r*_o_) between measures with a given reliability, for different correlations (*r*_h_) between the properties they aim to measure. The vertical dashed line indicates the attenuation of a 0.3 correlation for different levels of reliability. The horizontal dashed line indicates effect sizes which are attenuated to an observable correlation of 0.3 for different levels of reliability.

For example, de Haas et al. (2016) and de Haas and Schwarzkopf (2018a) recently argued that face inversion effects are driven by retinotopic tuning biases. A potential way of testing this hypothesis would be to probe a correlation between the corresponding neural and behavioral effects across observers. However, these authors did *not* use this type of strategy, because their measures did not reliably capture individual differences. **Figure 1A** shows the individual magnitude of the Thatcher illusion (Thompson, 1980) for 36 observers in de Haas and Schwarzkopf (2018a). The illusion was highly robust – every observer showed the effect for both, odd and even trials (all data are in the upper right quadrant). At the same time, the inter-individual *variance* was highly inconsistent (*r* = 0.20). That is, even though any two observers consistently show an effect greater than zero, the *difference between them* is not reliable.^1^

Compare this to **Figure 1B**, showing individual differences in proneness to the sound-induced flash illusion (Shams et al., 2000). Individual difference in this cross-modal measure were reliable across visual field locations (*r* = 0.84) and negatively correlated with the size of primary visual cortex (de Haas et al., 2012).

Typically, *a priori* knowledge about the reliability of *brain* measures is rather limited. The reliability of neural measures depends on a range of factors including participant state, imaging method, the amount of data collected per participant, hardware, acquisition parameters, experimental design, preprocessing, and analysis pipelines. A thorough review of these factors is beyond the scope of the current manuscript [for an overview regarding fMRI, including pointers to optimization tools see (Bennett and Miller, 2010)]. However, we note a few general trends: the reported test–retest reliability of morphological MRI estimates is very good, with typical intra-class correlations (ICCs) > 0.8 (Madan and Kensinger, 2017). The reliability of functional measures can vary substantially for both, electrophysiology (McEvoy et al., 2000; Martín-Buro et al., 2016) and MRI (Plichta et al., 2012; Termenon et al., 2016). For example, Brandt et al. (2013) report ICCs < = 0.4 for BOLD signal magnitudes in novelty encoding paradigms, whereas van Dijk et al. (2016) reported voxel-wise test–retest reliabilities > 0.8 or > 0.9 for the individual layout of visual field maps. Generally, fMRI reliability is higher for motor and sensory tasks compared to those involving higher cognition, and for block designs compared to event related ones (Bennett and Miller, 2010).

The importance of reliable measures has been pointed out before (Dubois and Adolphs, 2016; Mollon et al., 2017; Hedge et al., 2018). In the context of brain–behavior correlations, Vul et al. (2009) prominently highlighted that measurement reliability limits observable correlations. Following Spearman (1904), the observable correlation *r*_o_ for a given ‘true’ or hypothesized brain–behavior correlation *r*_h_ is attenuated by the geometric mean of the corresponding measurement reliabilities (*rel*_brain_ and *rel*_behav_):

(1) $$r_{o}=r_{h}*\sqrt{{\mathrm{rel}}_{\mathrm{brain}}*{\mathrm{rel}}_{\mathrm{behav}}}$$

**Figure 1C** illustrates the effect of this for different effect sizes and levels of reliability ($\sqrt{{\mathrm{rel}}_{\mathrm{brain}}*{\mathrm{rel}}_{\mathrm{behav}}}$). Researchers undertaking power calculations have to decide for a minimum effect size their study should be sensitive for. They may decide that a biologically meaningful effect implies a minimum of ∼9% shared variance and therefore aim for adequate power (>85%) to detect brain–behavior correlations > = 0.3. However, if the measures used have limited reliability, this has to be taken into account. Adequate power for an observable effect size *r*_o_ = 0.3 will correspond only to (much) stronger biological effects *r*_h_ if they are attenuated by unreliable measures (the horizontal dashed line in **Figure 1C**). Even a relatively moderate lack of reliability will result in a noticeable drop in power. Using measures with a reliability of 0.7, sensitivity for observable effects *r*_o_ = 0.3 would translate to biological effects *r*_h_ > 0.43. Conversely, preserving adequate power for ‘true’ effects *r*_h_ > 0.3 would require sensitivity for observable effect sizes *r*_o_ > 0.21 (vertical dashed line in **Figure 1C**). This implies an approximate *doubling* of the required sample size from *n* = 97 to *n* = 201.

Wherever possible, an investment in reliable measures seems more efficient than bringing a large sample with unreliable measures to the scanner. For novel measures, reliability should be quantified and reported (Dubois and Adolphs, 2016). This may have to be estimated *post hoc* for novel brain measures. But for *behavioral* measures it should be done *outside the scanner*. Prescreening can serve this purpose well and simultaneously be used for selective sampling.

## Sampling Selectively

The power to detect covariance depends on (true) in-sample variance. Prescreening allows maximizing variance through selective sampling. Here, I will briefly present the results of *ad hoc* simulations of this effect. The code is available at https://osf.io/hjdcf/ and interested readers can turn there to find more details and adjust parameters for their own power calculations.

To simulate the effect of a prescreening strategy, 10 bivariate populations were created by drawing 10^7^ normally distributed random ‘behavioral’ values *x* ∼ *N*(0,1). Corresponding ‘brain’ values (*y*) were simulated based on the normally distributed random variable *e* ∼*N*(0,1) and a defined observable brain–behavior correlation *r*_o_ (specific for each population and ranging from 0 to 0.9), such that

(2) $$y=r_{o}*x+\sqrt{1-r_{o}^{2}*e}$$

**FIGURE 2:**
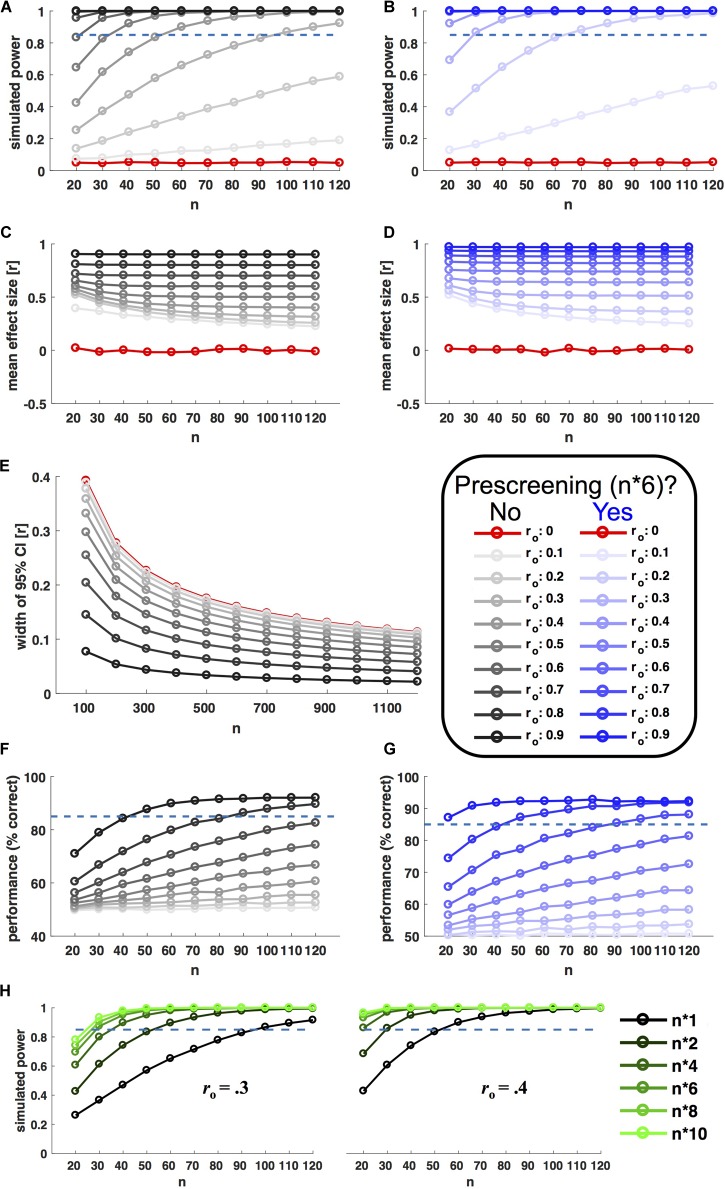
Selective sampling. Left hand plots in black ink show power simulations without prescreening; right hand plots in blue show corresponding results for prescreening with selective sampling of extreme groups. Selective samples were drawn based on behavioral measures from a prescreening group six times the size of *n* (extreme groups for **B,D**, even sampling for **G**). In each panel, ink saturation indicates the simulated effect size on the population level, as shown in the inset (observable correlation *r*_o_). Red ink indicates simulation results for zero-effects (*r*_o_ = 0). All *x* axes indicate sample size *n* (10.000 random samples drawn for each level). **(A,B)** Shows power, i.e., the fraction of samples showing a statistically significant correlation (*P* < 0.05). **(C,D)** Shows corresponding effect sizes (mean of observed significant correlations). **(E)** Shows the (inverse) precision of effect size estimates (width of 95% confidence interval) as a function of observed effect size and sample size. **(F,G)** Shows the accuracy of a model selection procedure aiming to distinguish between linear and non-linear relationships based on simulated data (see main text for details). **(H)** Shows the power boost afforded by different prescreening factors, for two different observable effect sizes (*r*_o_ = 0.3/0.4, as shown). The prescreening factor corresponds to the size of the prescreening sample divided by the final *n*, as shown in the legend to the right.

Results of *y* were only accepted if they correlated with *x* by *r*_o_ within a tolerated error margin of 0.01 (otherwise the procedure was repeated). From each of the resulting populations, 10.000 random samples with *n* observations were drawn with replacement (*n* ranging from 20 to 120 in steps of 10). For each level of *r*_o_ and *n*, Power was estimated as the fraction of the 10.000 samples showing a significant brain–behavior (Pearson) correlation (*P* < 0.05). **Figure 2A** shows the relationship between power, sample size and *r*_o_. In line with analytic predictions and recent recommendations (Dubois and Adolphs, 2016), researchers would indeed have to scan about 100 participants in order to achieve good power (>85%) to detect moderate observable effect sizes (*r*_o_ > 0.3).

**Figure 2B** shows the results of the same simulation incorporating prescreening. Here, for each sample size, behavioral measures (*x*) were first drawn for a larger prescreening sample, six times the size of *n*. Brain–behavior (Pearson) correlations were only probed in a subsample, for which *n* participants with extreme behavioral values were selected from the prescreening sample (n/2 participants with the lowest and highest prescreened values in *x*, respectively). This strategy resulted in a remarkable power boost (**Figure 2B**). With prescreening, good power (>85%) to detect *r*_o_ > 0.3 could be achieved with an in-scanner sample size of *n* = 30. Importantly, this power boost was limited to true effects. For *r*_o_ = 0 the nominal false alarm rate of *P* = 0.05 was preserved. **Figure 2H** shows corresponding results for further prescreening factors (i.e., multiples of *n* other than six).

The power boost afforded by prescreening does not come for free. To achieve comparable power to *n* = 100 at *n* = 30, the simulated researcher prescreened 180 participants. But the higher cost of neural compared to behavioral testing should typically render this a sensible choice. These factors can vary, but it is worth considering an example.

In the author’s experience, many behavioral experiments can be done in an hour, and the booking time for a typical fMRI session is 2 h. The standard fee for participant reimbursement at his current institution is 8€/h and that for booking a research MRI machine in Germany is 150€/h^2^. Assuming these numbers, we can calculate the price tag of improving a traditional, low-powered study (*n* = 30) to a well-powered one using different strategies. Prescreening in this example would require additional behavioral testing of 150 participants, which works out to additional costs of 150^∗^1h^∗^8€/h = 1.200€. A similar level of power could be achieved by testing *and scanning* 70 additional participants, which would work out to 70^∗^(1h^∗^8€/h + 2h^∗^(8€/h + 150€/h)) = 22.680€. Remarkably, prescreening achieves a similar power boost as the recommended increase in sample size *at ∼5% of the cost*.

I expect this estimate to be a conservative one. The assumed cost of scanning is a fraction of that charged in many centers outside Germany. The example also left aside all staff costs. A single student can typically do behavioral testing, while most neuroimaging facilities will require at least two professionally trained operators. Likewise, the analysis of neuroimaging data requires specialized training and can be more time consuming than that of behavioral experiments. Researchers and funding agencies are encouraged to do their own calculations, adjusting parameters as needed.

Note that error in this simulation was captured by the error term $\sqrt{1-r_{o}^{2}*e}$, which negatively scales with *r*_o_ (Eq. 2). The observable correlation *r*_o_ in turn depends on the shared variance between ‘true’ behavioral and neural traits, *r*_h_, as well as on measurement errors attenuating this relationship (Eq. 1). Expanding *r*_o_ to directly enter measurement error into the simulation yields identical results (also see section “Counterargument 3: Real Data May Be ‘Nastier’ Than Simulations” below). But specifying *r*_o_ in a separate, first step seems closer to practical purposes.

To consider an example: A researcher hypothesizes that retinotopically determined V1 surface area negatively correlates with individual proneness to the sound induced flash illusion (c.f. de Haas et al., 2012). First, she has to decide on a minimum ‘true’ effect size she considers biologically meaningful and the level of power she wants to achieve for this effect (let’s say 85% power for *r*_h_ > 0.36). Next she has to estimate the reliability of the corresponding behavioral (*rel*_behav_ = 0.84) and neural measures (*rel*_brain_ = 0.82) – which in this case could be done using previous literature (de Haas et al., 2012; Schwarzkopf and Rees, 2013). Based on these values (and following Eq. 1), she can now determine the minimum *observable* correlation she aims for as *r*_o_ > 0.3. Entering this into the power simulation code^3^, the researcher finds that she can achieve > 85% power, e.g., by scanning *n* = 30 extreme participants from a behavioral sample of n^∗^6 = 180 participants, or by scanning *n* = 40 extreme participants from a behavioral sample of n^∗^4 = 160 (**Figure 2H**). Based on available resources and costs, she decides for the former approach and tests proneness to the sound-induced flash illusion in 180 participants. After confirming *rel*_behav_ in this sample, she invites the 36 participants with highest and lowest proneness values back for a retinotopy scan (18 from either tail). The researcher scans the first 15 participants from either group accepting this invitation. This approach anticipates a dropout up tp 20% among those re-invited for scanning and according to supplementary simulations leaves the afforded power boost virtually unchanged (**Supplementary Figure 1C**).

## Caveats and Counterarguments

### Conterargument 1: Sampling Extreme Groups Will Yield Inflated Estimates of Effect Sizes

Yes. Researchers applying prescreening should be aware of this and highlight it in their publications. The application of correlation measures across extreme groups is well-established (Preacher, 2015) and more powerful than group comparisons (e.g., via *t*-tests). This is because correlation measures take into account the variance within as well as between extreme groups (Alf and Abrahams, 1975). At the same time, the increase in variance achieved through selective sampling inflates the size of (non-zero) correlations.

**Figures 2C,D** shows average effect size estimates with and without prescreening for the power simulation shown in **Figures 2A,B**. Effect size estimates are inflated in both cases, because they are based on significant results only [the simulation assumes a ‘ file drawer problem’ (Simonsohn et al., 2014)]. But the degree of inflation is larger for selective sampling. It will be especially important to take this into consideration for meta-analyses.

However, inflation is limited to true effects; for *r*_o_ = 0, the average effect size stays zero. Prescreening preserves a low false positive rate, while affording a high probability of detecting true effects. Just as for increased sample sizes, this results in a high positive predictive value of significant findings. That is, a significant result has a higher probability of reflecting a true effect when the study was well-powered – regardless of whether that power was achieved through prescreening or increased sample sizes.

Selective sampling is an efficient strategy for *detecting* (or rejecting) brain–behavior correlations. It is not suitable for precise estimates of their size or the parameters of a predictive model (c.f. Bzdok et al., 2018). Whenever precise effect size estimates are of interest, researchers should be aware that a sample size of *n* = 100 could be *far* too small^4^. The width of 95% correlation confidence intervals decreases with *n*, but only slowly. As shown in **Figure 2E**, for moderate effect sizes (*r*_o_ = 0.3) this measure of imprecision is still > 0.1 at *n* = 1000. In practice, precise effect size estimates will be limited to very strong effects or large-scale initiatives like the Human Connectome Project^5^ or UK Biobank^6^.

Finally, the description of a population parameter requires a well-defined population (Henrich et al., 2010; Smith and Little, 2018). Scanning an extremely large student sample would still yield a biased estimate of any population other than that of students. Researchers aiming at *representative* sampling can adapt prescreening to this end (see below).

### Counterargument 2: Sampling Extreme Groups Can Conceal Non-linear Brain–Behavior Relations

Yes. The extreme-group strategy proposed here aims at detecting (quasi-)linear relationships. Researchers aiming to compare different models should optimize their sampling strategy accordingly.

**Figures 2F,G** show the results of a simulation of ‘true’ linear and non-linear brain–behavior relationships. This simulation followed three steps. First, it drew random behavioral data (*x*) from a normal distribution *x* ∼*N*(0,1). Second, idealized brain predictions (*y*_h_) were generated, 50% of which perfectly corresponded to the model:

(3) $$y_{h}=x$$

and 50% of perfectly corresponded to the model

(4) $$y_{h}={\left(x+3\right)}^{2}$$

Importantly, a third step added brain measurement *noise*, which was manipulated to be comparable for both models and to the power calculations above. Specifically, brain measures (*y*) were simulated as random data linearly correlated to the respective model predictions (*y*_h_) with *r*_o_. In this way, 10.000 samples were drawn for both models at each level of *r*_o_ and sample sizes ranging from *n* = 20 to *n* = 120.

For each sample, the simulation aimed to distinguish linear from non-linear relationships by fitting 1^st^ and 2^nd^ order polynomials. The ‘wining’ model for each sample was chosen based on the Akaike Information Criterion (Akaike, 1974). As can be seen in **Figure 2F**, the proportion of correct model choices (unsurprisingly) increases as a factor of *r*_o_ and *n*. Note that adequate sensitivity for model comparison required very large sample sizes and/or strong effects.

To evaluate the usefulness of prescreening in such a scenario, the simulation was repeated for behavioral prescreening with n^∗^6. The sampling strategy was adjusted to the needs of model comparison. Instead of sampling the tails of the prescreening sample, the algorithm aimed at choosing a subsample that covered the behavioral range of the prescreening sample as evenly as possible (see online code for details). **Figure 2G** shows that this strategy significantly enhanced the sensitivity to discriminate non-linear from linear relationships.

### Counterargument 3: Real Data May Be ‘Nastier’ Than Simulations

Yes. The simulations presented here assume normally distributed data and random errors. Real data may be less well-behaved. However, it is not clear *a priori* that this would pose more of a problem for the prescreening compared to a full-sample approach. Moreover, prescreening samples enable informed hypotheses regarding potentially problematic aspects of the data.

A particularly relevant example of problematic data is that of *heteroscedastic measurement error*, scaling with the latent variable. In this scenario, extreme groups will be affected by particularly low and high measurement errors, respectively. Importantly, supplementary simulations confirm the robustness of prescreening in this situation. Prescreening and selective sampling preserved nominal false positive rates and a strong power boost, even for a population with strongly heteroscedastic measurement error (**Supplementary Data** and **Supplementary Figure 1**).

### Counterargument 4: Extreme Groups May Be Special

Yes, but it is important to spell out what that means.

It may refer to the *relationship* of a behavioral and a neural trait not following a uniform, linear model across the entire distribution. In such cases, the underlying linear model is inappropriate, regardless of the sampling approach. See Counterargument 2 for prescreening in the context of non-linear model comparisons.

Alternatively, the argument may refer to *measurement error*. Even random measurement errors will correlate with observed values. That is, extreme measurements will partly reflect extreme errors (and more so for less reliable measures). This biased error sampling causes regression to the mean (Barnett et al., 2004). Importantly, extreme observed values *also* reflect true extreme values (at least for measures with non-zero reliability). This is the source of the power boost afforded by prescreening. Additional simulations show that the elevation of error variance in extreme groups is much smaller than that of true variance, even for reliabilities as low as 0.3. For a given *r*_o_, prescreening causes virtually identical power boosts, regardless of the constituting *r*_h_ and reliabilities (**Supplementary Data** and **Supplementary Figure 2**). Another, more specific worry is that of measurement errors scaling with the latent variable. See Counterargument 3 and **Supplementary Material** for the robustness of prescreening to heteroscedastic errors.

Finally, *confounding factors* may be especially pronounced in behavioral extreme groups. Statistical control for confounding factors can be difficult if they have to be estimated with noisy measures (Westfall and Yarkoni, 2016). Behavioral prescreening is an excellent tool to estimate the magnitude of confounding factors as well the associated measurement errors across the distribution. If parts of the distribution are particularly affected by confounding factors or by measurement noise for the corresponding estimates, this can motivate a sampling strategy targeting more robust cases (which may or may not coincide with extreme groups, c.f. Counterargument 2).

### Counterargument 5: Prescreening Should Be Combined With Large Samples Rather Than Pitted Against Them

Not necessarily.

Researchers interested in precise effect size estimates will indeed need four-figure sample sizes, unless the effects they study are unusually large. However, the aim of many brain–behavior studies is arguably more humble. Researchers often are interested in testing the hypothesis that there is *some* relationship between a given neural and a behavioral measure which is captured well-enough by a linear model to be relevant in the context of their theory (e.g., *r*_o_ > 0.3). Testing this hypothesis can be a valuable first step, even though (for affirmative cases) it certainly should not be the last (Smith and Little, 2018). Most importantly, detection studies can only give valid pointers to relevant effects if they are well-powered.

Prescreening *can* ensure adequate power with small sample sizes in the scanner, even for moderate effects (e.g., >85% power for *r*_o_ > 0.3 at *n* = 30). Researchers combining this approach with individually consistent measures can be confident in the detection power and replicability^7^ of their studies (**Figure 2B**). The savings afforded by this strategy relative to a blanket increase of sample sizes should routinely be around a factor 20 or higher. Given the limited resources available to neuroscience, these savings would translate to a larger number of well-powered detection studies.

### Counterargument 6: Prescreening Precludes the Reuse of Data for Unrelated Research Questions

Yes (usually). Neuroimaging experiments can produce data that are highly specific to an underlying research question, like the retinotopic specificity of visual cortex responses to illusory contours (de Haas and Schwarzkopf, 2018b). However, most experiments yield at least some data that can potentially be ‘recycled’ for entirely unrelated questions (like structural and diffusion weighted scans, or retinotopic maps). Prescreened samples are inherently biased and as such not ideally suited for such recycling. Does that mean the savings afforded by pre-screening are only short-term?

Not in the author’s opinion. The best sources of ‘general purpose’ data are public datasets like the aforementioned Human Connectome Project. These are truly large scale, representative and include extensive test batteries. This will typically not be the case for data from individual experiments. Consider a researcher investigating the relationship between V1 surface area and the individual strength of contextual size illusions (Schwarzkopf et al., 2011). Several years later, the same researcher may become interested in the question whether V1 surface area varies with fluid intelligence. It is true that prescreening for the initial experiment would yield a smaller and potentially biased dataset for the second question. But *not* to prescreen would only marginally improve the situation. The typical sample would still be anything but representative and most likely miss crucial data. At the same time a high quality public dataset is readily available (Benson et al., 2018). Division of labor appears a far more efficient approach for human neuroscience: ‘General purpose’ data are acquired in dedicated large-scale studies, while hypotheses requiring more specific experiments are tested in studies optimized to *that* end. The latter type can profit from prescreening.

### Counterargument 7: Prescreening Sometimes Is Not Feasible

Yes. For instance, it can be more efficient to scan every participant if the recruitment process itself is costly (as for some special populations). In general, the cost ratio of behavioral and neural testing will vary with the type of behavioral measure. The example calculation given above assumed a simple, lab-based psychophysics or eyetracking experiment. The potential advantage of prescreening will be even more pronounced for questionnaires, or tasks that can be completed online. At the other end of the spectrum are resource-intensive behavioral tasks, like reverse correlation techniques requiring 10s of 1000s of trials (Gosselin and Schyns, 2003), which will render the prescreening cost-advantage small or even absent.

Furthermore, for some behavioral tasks it may be impossible to estimate the reliability of between subject variance, for instance because they require participants to be naïve and cannot be repeated (like some measures of perceptual learning). However, such scenarios will typically call for an entirely different research design. Measures that cannot be tested for the reliability of between-subject variance are unsuitable for brain–behavior correlations as such, not just for prescreening. Group level effects can be robust independently of this (**Figure 1A**) and should be the variable of interest in this type of situation.

## Conclusion

Like all of science, studies aiming at the detection of brain–behavior correlations depend on well-powered experiments, yielding replicable results. This perspective highlighted how this can be achieved with relatively small sample sizes in the scanner. Behavioral prescreening can achieve a power boost comparable to larger sample sizes at a fraction of the cost and without inflating the false positive rate.

Researchers investigating brain–behavior correlations should base their power calculations on a broader basis than sample size alone. The simulation code accompanying this perspective can be used to incorporate the boost afforded by prescreening. Researchers should also pay attention to the reliability of measures and adjust their power estimates for attenuation. Similarly, reviewers, editors and funding agencies should refrain from a simple but false heuristic of large in-scanner samples as a necessary or sufficient criterion for adequate detection power.

## Author Contributions

BdH: coded simulations, prepared figures, and wrote the paper.

## Conflict of Interest Statement

The author declares that the research was conducted in the absence of any commercial or financial relationships that could be construed as a potential conflict of interest.

## Footnotes

1 Note that this may partly be due to ceiling effects and a limited number of trials. Individual face inversion effects in other designs may vary more consistently (Rezlescu et al., 2017).

2 http://www.dfg.de/formulare/55_04/55_04_de.pdf

3 https://osf.io/hjdcf/

4 http://datacolada.org/20

5 http://www.humanconnectomeproject.org/

6 http://www.ukbiobank.ac.uk/

7 Note that replicability here refers to the detection question: experiments will give reliable answers to the question whether there is an effect whenever there is *no* effect (repetitions of the experiment will yield mostly negative results) or a true effect of a *magnitude they are well-powered for* (repetitions of the experiment will mostly yield significant results). Replicability in a stricter sense of near-identical effect size estimates is much harder to achieve (3.1). Both concepts are linked in the sense that more precise effect size estimates imply power for smaller effect sizes. That is, more precise effect size estimates reduce the range of true effects the experiment is *not* well-powered for.
