# Supplementary material for: How to Enhance the Power to Detect Brain–Behavior Correlations With Limited Resources
Source: Front Hum Neurosci. 2018 Oct 16;12:421. doi: 10.3389/fnhum.2018.00421 (PMC6198725; doi:10.3389/fnhum.2018.00421)
Supplement: Supplementary file 1 [file Data_Sheet_1.PDF]

Supplementary Material

How to enhance the power to detect brain-behavior correlations with limited resources

Benjamin de Haas

Correspondence: benjamindehaas@gmail.com

1 Supplementary Data

Supplementary simulations probed three concerns regarding the prescreening approach presented in the main manuscript. The first analysis tested the effects of participant dropout after behavioral prescreening (end of section 2 in the main manuscript). The second analysis tested the robustness of the approach to heteroscedastic measurement errors, scaling with the latent behavioral and neural variables (Counterargument 3 in the main manuscript). The third probed the robustness of prescreening to different levels of reliability. Here, we provide a short description of these simulations and present their results. For full details, readers are referred to the simulation code, which is publicly available at https://osf.io/hjdcf/.

1.1. SIMULATING DROPOUT

This analysis followed all details of the main simulation with the exception of the sampling procedure for extreme groups. Instead of sampling the most extreme n/2 cases, this variant sampled n*0.6 cases from either end of the prescreened distribution. In a second step, n/2 cases were randomly sampled from either extreme group to simulate a dropout rate of 20%.

1.2. SIMULATING HETEROSCEDASTIC MEASUREMENT ERROR

This analysis deviated from the main simulation in the way measurement errors were modelled. First, behavioral and neural measurement reliabilities were fixed to

$$rel_{\text{behav}} = rel_{\text{brain}} = .7 \quad \text{(S1)}.$$

Correspondingly, the correlation r_h between 'true' behavioral and neural scores was defined as

$$r_h = r_o / rel_{\text{behav}} \quad \text{(S2)},$$

and clipping the absolute value of $|r_\mathrm{h}| < 1$. The simulation then drew a population of 10^7 normally distributed true behavioral values $x_l \sim N(0,1)$. The corresponding true brain values y_t were computed as

$$y_l = r_h * x_l + \sqrt{1 - r_h{}^2} * e \quad \text{(S3)},$$

with the random variable $e \sim N(0,1)$. Crucially, these true values were then transformed into the observed values x and y with heteroscedastic measurement error as follows:

$$x = rel_{behav}{}^{0.4} * z(x_l) + \sqrt{1 - rel_{behav}{}^{0.8}} * e * 2p(x_l) \quad \text{(S4)},$$

with the random variable $e \sim \mathrm{N}(0,1)$; $z(x_l)$ denoting the z-value $(x_l\text{-}\mu(x_l))/\sigma(x_l)$; and $p(\mathrm{x}_l)$ denoting the cumulative density function at $z(x_l)$ (i.e. the area under the normal density curve from $-\infty$ to $z(x_l)$).

Accordingly, y_l was transformed into y as

$$y = rel_{brain}{}^{0.4} * z(y_l) + \sqrt{1 - rel_{brain}{}^{0.8}} * e * 2p(y_l) \quad \text{(S5)}.$$

The resulting measures x and y were estimates of x_l and y_l with strongly heteroscedastic measurement errors (Supplementary Figure 1A) and correlated with each other with the desired pre-specified level r_o on the population level (with r_o being limited to a maximum of 0.7, because r_h cannot exceed 1, equation S2). They were simulated for different levels of r_o and used for power simulations involving prescreening, following the same routines as the main simulation.

1.3. SIMULATING DIFFERENT LEVELS OF RELIABILITY

The purpose of this analysis was to probe, whether the power boost afforded by prescreening for a given level of r_o depends on the constituent contributions of reliabilities and r_h. Tests with low reliability will be more prone to biased error sampling, which may affect the efficacy of the approach.

This analysis was similar to 1.2. above, but fixed the observable correlation r_o to .3 and let reliability range from .3 to 1 in increments of .1 (with $rel_\mathrm{behav} = rel_\mathrm{brain}$). For each level of reliability, r_h was determined using equation S2. Next, true behavioral (x_l) and neural (y_l) scores were generated as following equation S3. The observed values x and y were then derived as:

$$x = rel_{behav} * x_l + \sqrt{1 - rel_{behav}{}^2} * e \quad \text{(S6)}$$

and

$$y = rel_{brain} * y_l + \sqrt{1 - rel_{brain}{}^2} * e \quad \text{(S7)}$$

both with the random variable $e \sim N(0,1)$.

Following the same routines as the main simulation, x and y were used for power simulations with and without prescreening (n*6). Power with and without prescreening was plotted for each level of reliability and across sample sizes. Additionally, we plotted the in-sample total and error variance for each level of reliability with and without prescreening (average across 10.000 samples with $n = 30$).

Results showed that power and the power for a given r_o was independent of the underlying r_h and reliabilities (Supplementary Figure 2). This result was replicated for an additional simulation fixing $r_o = .3$ and $r_h = .6$, while varying rel_{behav} (from .3 to 1 in steps of .1), which simultaneously determined rel_{brain} as:

$$rel_{brain} = \frac{\left(\frac{r_o}{r_h}\right)^2}{rel_{behav}} \qquad \text{(S8)}$$

(c.f. Equation 1). The results of this analysis were identical to those shown in Supplementary Figure 2 and can be replicated running the accompanying online code. Both results converge to show that power is a function of r_o, sample size and sampling strategy, but given r_o is independent of the underlying combination of r_h and reliabilities. In practice, researchers will of course aim to detect a given true effect r_h and r_o will dramatically depend on measurement reliability (equation 1 and Figure 1 in main manuscript).

2 Supplementary Figures

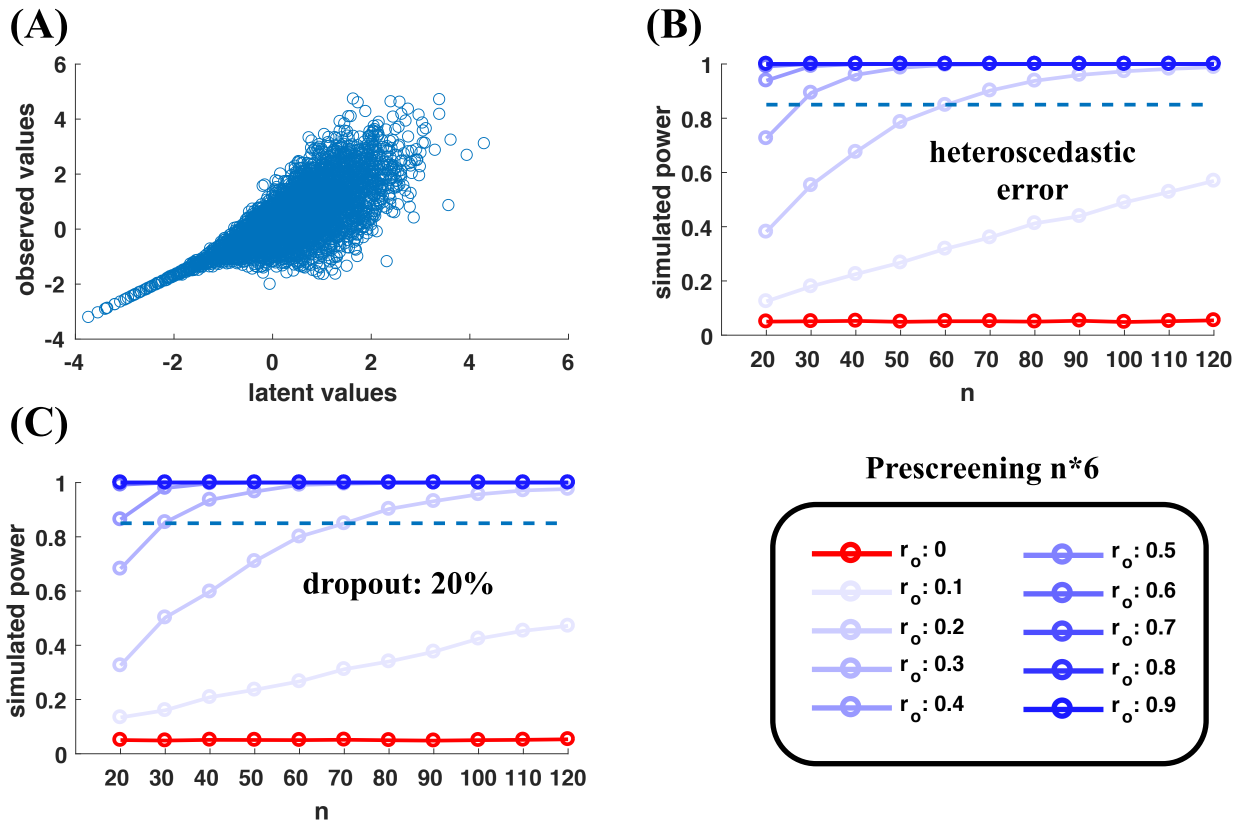

Supplementary Figure 1. Supplementary simulations of dropout and heteroscedastic error. Panel **A)** shows the strongly heteroscedastic error for 10^4 randomly sampled datapoints from a population of 10^7 latent (x_l) and observed (x) behavioral values, simulated according to equation S4. Panel **B)** shows the results of corresponding power simulations, based on observations with strongly heteroscedastic errors and using behavioral prescreening with a sample size of n*6. Panel **C)** shows simulated power for behavioral prescreening with n*6, assuming selective sampling of 0.6*n cases at either end of the distribution and a random drop out of 20% of those cases before correlation. Different levels of observed correlations r_o are indicated by saturation, as shown in the inset.

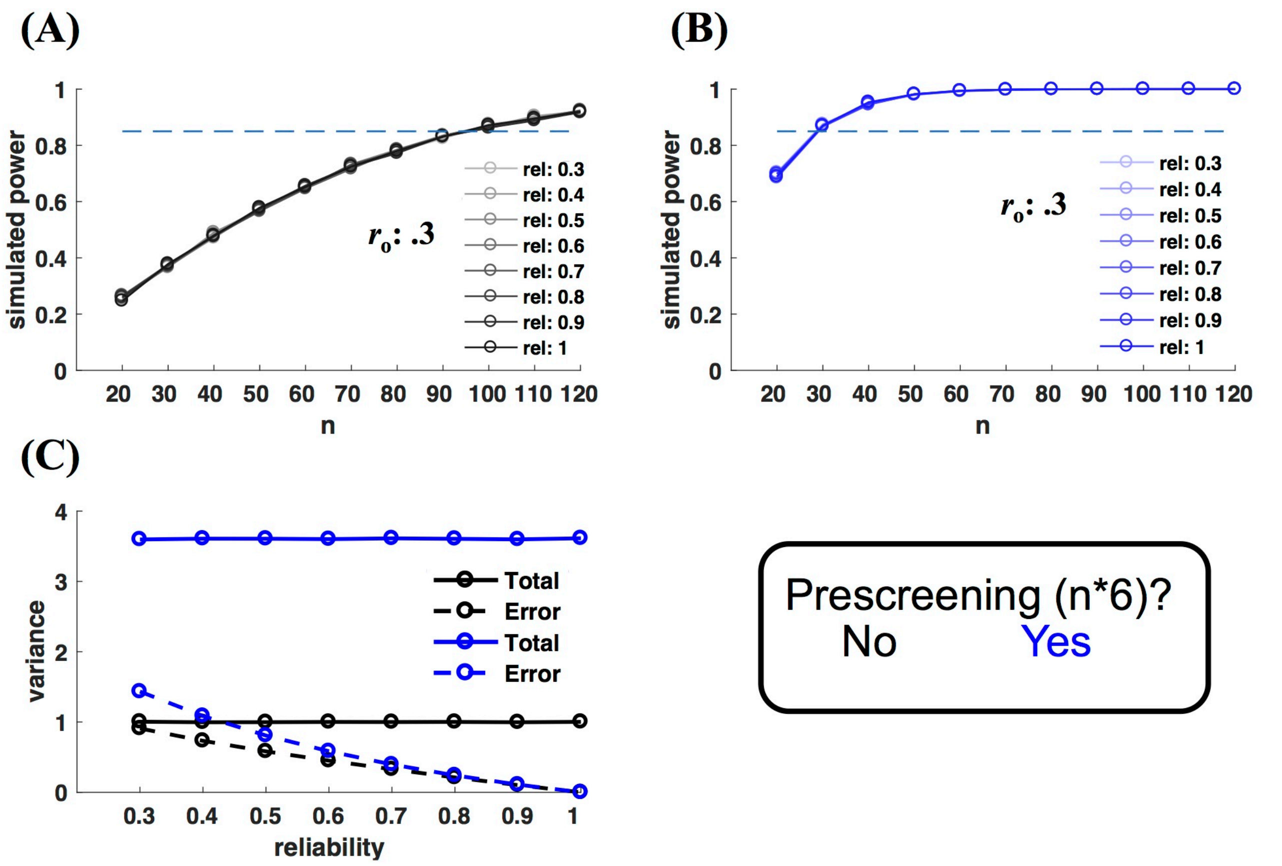

Supplementary Figure 2. Supplementary simulations of different levels of reliability. Panel **A)** and **B)** show simulated power without and with prescreening, respectively. The observable (population) correlation in both cases was fixed to .3, but the underlying reliability and true correlation varied as shown in the inset (true correlations varied inversely according to equation S2). Curves for different reliabilities are almost perfectly overlapping, showing that power depended on r_o, sampling strategy and sample size, but not on the combination of r_h and reliabilities underlying r_o. Note that researchers in practice will aim to detect a given r_h and therefore r_o and power will vary dramatically with measurement reliability (Figure 1 in the main manuscript). Panel **C)** shows levels of total and error variance for one measure (average across 10.000 samples with $n = 30$). Blue and black lines show in-sample variance with and without prescreening, respectively. Solid and dashed lines show total and error variance, respectively. Note that the regression error between measures will depend on r_h as well and that r_h inversely varies with reliability for a given r_o (equation S2). While total in-sample variance is constant across reliabilities, error variance decreases with reliability. For very low reliabilities, prescreening somewhat inflates error variance (reflecting biased error sampling for extreme groups). However, this effect is small compared to the total gain in variance.
